# Germline Mutation Analysis in Sporadic Breast Cancer Cases With Clinical Correlations

**DOI:** 10.3389/fgene.2022.820610

**Published:** 2022-03-09

**Authors:** Sadia Ajaz, Sani-e-Zehra Zaidi, Saleema Ali, Aisha Siddiqa, Muhammad Ali Memon

**Affiliations:** ^1^ Dr. Panjwani Center for Molecular Medicine and Drug Research (PCMD), International Center for Chemical and Biological Sciences (ICCBS), University of Karachi, Karachi, Pakistan; ^2^ Department of Human Genetics and Molecular Biology, University of Health Sciences, Lahore, Pakistan; ^3^ Atomic Energy Medical Centre (AEMC), Jinnah Postgraduate Medical Centre (JPMC), Karachi, Pakistan

**Keywords:** breast cancer, susceptibility, genomics, next-generating sequencing, candidate genes, Pakistani population

## Abstract

Demographics for breast cancers vary widely among nations. The frequency of germline mutations in breast cancers, which reflects the hereditary cases, has not been investigated adequately and accurately in highly-consanguineous Pakistani population. In the present discovery case series, germ-line mutations in twenty-seven breast cancer candidate genes were investigated in eighty-four sporadic breast cancer patients along with the clinical correlations. The germ-line variants were also assessed in two healthy gender-matched controls. The clinico-pathological features were evaluated by descriptive analysis and Pearson χ^2^ test (with significant p-value <0.05). The most frequent parameters associated with hereditary cancer cases are age and ethnicity. Therefore, the analyses were stratified on the basis of age (≤40 years vs. >40 years) and ethnicity. The breast cancer gene panel assay was carried out by BROCA, which is a genomic capture, massively parallel next generation sequencing assay on Illumina Hiseq2000 with 100bp read lengths. Copy number variations were determined by partially-mapped read algorithm. Once the mutation was identified, it was validated by Sanger sequencing. The ethnic analysis stratified on the basis of age showed that the frequency of breast cancer at young age (≤40 years) was higher in Sindhis (*n* = 12/19; 64%) in contrast to patients in other ethnic groups. Majority of the patients had stage III (38.1%), grade III (50%), tumor size 2–5 cm (54.8%), and invasive ductal carcinoma (81%). Overall, the analysis revealed germ-line mutations in 11.9% of the patients, which was not significantly associated with younger age or any particular ethnicity. The mutational spectrum was restricted to three genes: *BRCA1*, *BRCA2*, and *TP53*. The identified mutations consist of seven novel germ-line mutations, while three mutations have been reported previously. All the mutations are predicted to result in protein truncation. No mutations were identified in the remaining twenty-four candidate breast cancer genes. The present study provides the framework for the development of hereditary-based preventive and treatment strategies against breast cancers in Pakistani population.

## Introduction

Epidemiological studies have shown ethnic and geographic differences in breast cancer etiology. The increased susceptibility to breast cancers has been attributed to socio-economic, environmental, and genetic factors (Zeeshan et al., 2019). Hereditary breast cancers comprise a significant number. In the US population, these constitute 10–15% of the cases. There is paucity of data from low- to middle- income countries (LMIC) (Akarolo-Anthony et al., 2010; Jazayeri et al., 2015).

It has been estimated that half of all breast cancer cases occur in the 12% women who are at the maximum genetic risk (El Saghir et al., 2007). *BRCA1* and *BRCA2* are high penetrant breast cancer genes. These have been especially associated with hereditary breast and/or ovarian cancers. The mutations in these genes are considered to increase the life-time risks of breast cancer by 82% (Antoniou and Easton, 2006; Shiovitz and Korde, 2015). Other highly penetrant but rare genes include *PTEN, TP53*, *CDH1,* and *STK11.* Moderate penetrance genes, which increase the risk for breast cancer by twofold, include genes involved in DNA repair such as *ATM, BRIP1* (*BACH1*), *CHEK2*, and *PALB2*. Still other genes are considered to confer a low but significant risk for breast cancers (Walsh et al., 2017).

In Pakistani population, some studies have analyzed *BRCA1* and *BRCA2* mutations (Liede et al., 2002; Rashid et al., 2006; Moatter et al., 2011; Ahmad et al., 2012; Aziz et al., 2016; Rashid et al., 2016; Rashid et al., 2017; Torres et al., 2017), mainly through conventional methodologies. In case of other putative breast cancer susceptibility genes, scarce or no data is available from this region (Khaliq et al., 2000; Baig et al., 2011; Rashid et al., 2014; Baloch et al., 2016; Rashid et al., 2019). The introduction of next generation sequencing (NGS) including multi-gene testing necessitates re-assessment of the available information and generation of missing data.

Pakistani population comprises distinct ethnic groups. These include Sindhis, Balochis, Brahui, Makrani, and Parsis from Southern Pakistan. The other ethnicities Punjabis, Pathans, Hindko, Hazara, Kalash, Kashmiri and Burusho are from Northern Pakistan (Qamar et al., 1999; Khaliq et al., 2000). The data for these genetically distinct ethnic groups has not been incorporated in the few relevant regional-based publications on the breast cancers. The location of present study is a metropolitan city, situated in Southern Pakistan. The population comprises multiple ethnic communities from all over Pakistan. In addition, a self-defined Urdu-speaking ethnicity, comprising immigrants from India, is also a major group residing in the city.

The present study investigates molecular epidemiology of breast cancers from Southern Pakistan. It is the first such report from this region. The study investigates the inherited contribution to breast cancers by next-generation sequencing. A panel of twenty-seven breast cancer-associated candidate genes, has been analysed in breast cancer patients belonging to genetically distinct Pakistani ethnic groups.

## Materials and Methods

### Patients

Participants in the present study included 82 females and 02 males from Southern Pakistan. In total, 84 diagnosed cases of breast cancer were included in the study. The participants visited a tertiary care hospital: the Atomic Energy Medical Centre (AEMC), Jinnah Post-Graduate Medical Centre (JPMC), Karachi, Pakistan, from July 2016–July 2017. The patients were treated for primary invasive breast cancer post-mastectomy.

As the present study was carried out for the discovery purposes at the preliminary stage, all the clinically diagnosed primary breast cancer cases were included regardless of the age and/or family history. All the participants signed an informed consent form. Independent ethical review boards of the participating institutions approved the protocol.

### Demographic and Clinico-Pathological Information

Patients were interviewed about their family history of cancers (breast cancer and/or any other cancer), ethnicity, age at menarche and menopause (if applicable), and gynecological and obstetrics history. The medical records were reviewed for breast cancer diagnosis, staging, grading, and tumour size. According to the clinical reports, staging was carried out based on American Joint Committee on Cancer (AJCC), while grading was performed with Nottingham Histologic Score System based on Scarff-Bloom-Richardson Grading System.

### Genomics

The participants contributed 5–8 ml of blood samples for DNA extraction. Germ-line DNA was extracted from the patient’s WBCs by standard phenol-chloroform method (Sambrook and Russell, 2001). DNA was quantified spectrophotometrically (Beckman Coulter™, DU^®^ 530). Sufficient DNA was available for 84 subjects. BROCA, a targeted capture and multiplexed massively parallel sequencing gene panel assay was performed (Walsh et al., 2017). Briefly, it is a next-generation sequencing assay on Illumina Hiseq2000 with 100bp read lengths. The copy number variations (CNVs) were determined by partially-mapped read algorithm (Nord et al., 2011). It enables detection of all types of mutations for candidate and established breast cancer genes. Twenty-seven genes, which are highly associated with breast cancers, were investigated in the project: *BRCA1*, *BRCA2*, *TP53*, *ATR*, *BARD1*, *BRIP1*, *FAM175A*, *FANCM*, *GEN1*, *MRE11A*, *NBN*, *RAD51B*, *RAD51C*, *RAD51D*, *RECQL*, *RINT1*, *SLX4*, *BAP1*, *PALB2*, *PTEN*, *STK11*, *XRCC2*, *ATM*, *CHEK1*, *CHEK2*, *CDH1*, and *CTNNA1*.

### Validation of Mutations

After the NGS investigations, the identified mutations were validated by Sanger sequencing. Previously published protocols were used for the amplification of exons (Friedman et al., 1994; Friedman et al., 1997; Olivier et al., 2002) followed by standard method for Sanger sequencing.

### Statistical and Bio-Informatic Analysis

Data were entered, encoded and analysed using SPSS, version 17.0 (IBM™, United States). Breast cancer cases in the present study were grouped into three categories: age at sampling (≤40years vs >40 years); receptor (estrogen, progesterone, and HER2/Neu) status; and ethnicities. Descriptive analysis was carried out for the evaluation of demographics and clinico-pathological features. Groups were compared by Pearson χ^2^ test of independence for the clinico-pathological features: tumour size, grade, and stage. The p-values <0.05 were considered to be statistically significant.

The mutations were compared against BIC (BIC, 2019) and ExAC (ExAC, 2019) databases for novelty.

## Results

### Demographic Analysis

Total study included 84 breast cancer patients, with a diagnosis of primary breast cancer. Patients’ demographics are shown in Table 1. In the present cohort, majority of the breast cancer cases belonged to Urdu-speaking (25%) and Sindhi (24%) ethnicities. The frequency of breast-cancer cases among young patients (≤40 years) was higher in Hindko (*n* = 4; 75%) and Sindhi (*n* = 19; 64%) ethnicities, in contrast to other ethnic groups. Among other ethnic groups, the numbers of breast cancer cases in older patients (i.e., > 40 years) exceeded those who were ≤40 years.

**TABLE 1:** Patients’ demographics.

Sr No	Characteristic	Total	Patients’ age ≤40 years	Patients’ age >40 years
1	Total unrelated patients	84	35 (42%)	49 (58%)
	Female patients	82	35 (43%)	47 (57%)
	Male patients	02	00 (0%)	02 (100%)
2	Mean Age_samp_ (years)	44 (20–70)	35 (20–40)	51 (41–70)
4	Ethnicity^a^			
	Baloch	08	02 (25%)	06 (75%)
	Hindko	04	02 (50%)	02 (50%)
	Punjabi	05	02 (40%)	03 (60%)
	Pathan	9	03 (33%)	06 (67%)
	Sindhi	19	12 (63%)	07 (37%)
	Urdu-Speaking	19	09 (47%)	10 (53%)
	Others	11	02 (18%)	09 (82%)

a Ethnicities were unknown for 09 patients.

### Clinicopathological Evaluation

Pathology records were sought for the patients. Data were available for 70.9, 95, and 82.5% patients in case of tumour stage, grade and hormonal status, respectively. Among patients with available pathology data, the distribution of tumor stage was 5% Stage I, 33% Stage II, 56% Stage III, and 6% Stage IV. The distribution of tumour grade was 1.2% Grade I, 48.7% Grade II, and 50% Grade III. Overall, the distribution among stages and grades (low vs high) varied significantly (*p* <0.01). In case of tumours with available hormone profiles, 26% were triple negative (TNBC). Supplementary Table S1 lists the available clinicopathological information.

### Novel Germ-Line Mutations

A total of 84 samples were analyzed based upon sufficient DNA quantity. Genomic analysis of known breast cancer genes revealed that 11.9% (10/84) patients carried an unambiguously pathogenic germline mutation in three genes: *BRCA1*, *BRCA2*, and *TP53* (Table 2)*.* Novel germ-line mutations were identified in seven patients (3 in *BRCA1*, 3 in *BRCA2*, and 1 in *TP53*) (Table 2 and Supplementary Figure S1). The predicted outcome of all the identified mutations is protein truncation.

**TABLE 2:** Summary of germline mutations identified in *BRCA1*, *BRCA2*, and *TP53* genes in Pakistani breast cancer patients.

Sr No	ID	Histology^a^	Sex	Age group	Chromosome	Position start hg 37	Gene	C. DNA	Protein	Novel^b^ mutation
1	09	pBC (IDC)	F	≤40	13	32,914,299	*BRCA2*	c.5807delTGTC (ex11)	1936fs	yes
2	38	pBC (IDC)	F	>40	17	41,276,045	*BRCA1*	c.68_69del (ex2)	23fs	no
3	45	pBC (IDC)	F	≤40	17	41,244,237	*BRCA1*	c.3311delA (ex11)	K1104fs	yes
4	47	pBC (IDC)	F	≤40	17	41,253,030	*BRCA1*	del exons 5–7	K45fs	yes
5	63	pBC (IDC)	F	≤40	17	7,578,212	*TP53*	c.637C > T (ex6)	R213X	no
6	71	pBC (IDC)	F	>40	13	32,954,023	*BRCA2*	c.9090delA (ex23)	T3030fs	yes (germline)
7	73	pBC (IDC)	F	≤40	17	41,197,784	*BRCA1*	c.5503C > T (ex24)	R1835X	no
8	84	pBC (IDC + DCIS)	F	≤40	17	41,245,795	*BRCA1*	c.1753G > T (ex11)	E585X	yes
9	88	pBC (IDC)	F	>40	13	32,914,134	*BRCA2*	c.5642delAATC (ex11)	1881fs	yes
10	93	pBC (IDC)	F	>40	17	7,579,415	*TP53*	c.272G > A (ex4)	W91X	yes (germline)

a pBC: primary Breast Cancer; IDC: invasive ductal carcinoma; DCIS: Ductal Carcinoma *in situ*.

b Novelty was identified by comparing with BIC, and ExAC databases.

### Burden of Mutations in Breast Cancer Genes

Genomic analysis of known breast cancer genes showed that 15.3% (6/39) of patients with age ≤40 years, whereas 8.5% (4/47) of patients with age >40 years carried a definitive pathogenic germline mutation in three identified genes (Table 3).

**TABLE 3:** Distribution of germ-line mutations in breast cancer patients. Data has been stratified on the basis of age, hormone receptor status, and ethnicities.

Sr No	Characteristic	Total carriers	Frequency of mutation carriers		
			Any Gene	BRCA1/2	TP53
1	Total patients with mutations	10	0.12 (10/84)	0.1 (08/84)	0.03 (02/84)
2	Age at sampling (Age_samp_)				
	Age_samp_ ≤ 40 years	6	0.15 (6/39)	0.13 (5/39)	0.03 (1/39)
	Age_samp_ > 40 years, positive FH	4	0.08 (4/51)	0.06 (3/51)	0.02 (1/51)
3	Tumour hormone receptors				
	TNBC	4	0.23 (4/17)	0.23 (4/17)	0
	Non TNBC	3	0.06 (3/49)	0.02 (1/49)	0.04 (2/49)
	Unknown	3	0.3	0.2	0.1
4	Ethnicity				
	Baloch	0	0	0	0
	Hindko	2	0.5 (2/4)	0.5 (2/4)	0
	Punjabi	1	0.13 (1/8)	0.13 (1/8)	0
	Pathan	0	0	0	0
	Sindhi	2	0.11 (2/19)	0.06 (1/19)	0.06 (1/19)
	Urdu-Speaking	3	0.15 (3/20)	0.15 (3/20)	0
	Others	0	0	0	0
	Unknown	2	0.18 (2/17)	0.18 (2/17)	0

Among younger patients, 13% (5/39) carried a damaging mutation in *BRCA1* and *BRCA2* while 3% (1/39) carried a germ-line mutation in *TP53*. In the older patients, 5.8% (3/51) harboured a germ-line mutation in *BRCA1* and *BRCA 2,* whereas the frequency of germ-line mutation in *TP53* was 2% (1/51). Stratified analysis showed that the highest frequency of germ-line mutations in the investigated genes was in Hindko group (50%), followed by Urdu-speaking (15%), Punjabi (13%), and Sindhi (11%) ethnicities.

Among patients with known tumour hormone receptor status, 23% (4/17) TNBC patients carried a pathogenic germline mutation in *BRCA1*/*2* genes (2 in *BRCA1* and 2 in *BRCA2*).

Each identified mutation was present in only one family and no recurrent mutation was found in the present cohort. In the samples from two male patients, which were included in the study, no mutation in the selected genes was observed.

Further, no pathogenic germ-line variants were identified in the DNA from control samples.

## Discussion

Breast cancer is the most frequently diagnosed malignancy among women and the leading cause of cancer-related mortality in developing countries (Ferlay J et al., 2018). It is estimated that globally 1 in 6 women is diagnosed with breast cancer and 1 in 8 women has invasive form of the cancer. However, substantial differences have been observed in breast cancer indices across different populations (Bray et al., 2015). The average age in Caucasians is 63 years. In the present study we report an average of 44.4 years, whereas from the same region it is reported in the range of 50–53 years from India, and 46–49 years from Iran, respectively (Ahmadi et al., 2015; Desmond et al., 2015; Mannan et al., 2016; Howlader et al., 2017).

Majority of familial aggregation in breast cancers is unexplained. Environmental factors are unlikely to explain the residual familial clustering (El Saghir et al., 2007). Among Caucasians, with the increasing application of next generation sequencing in the clinical setting, an upward trend (26%) in reports of hereditary breast and/or ovarian cancers is observed (Antoniou and Easton, 2006). A number of large scale studies report germ-line mutational frequencies ranging from 9 to 26% in critical breast cancer genes (Stratton and Rahman, 2008; Castéra et al., 2014; Buys et al., 2017; Eliade et al., 2017).

**FIGURE 1:**
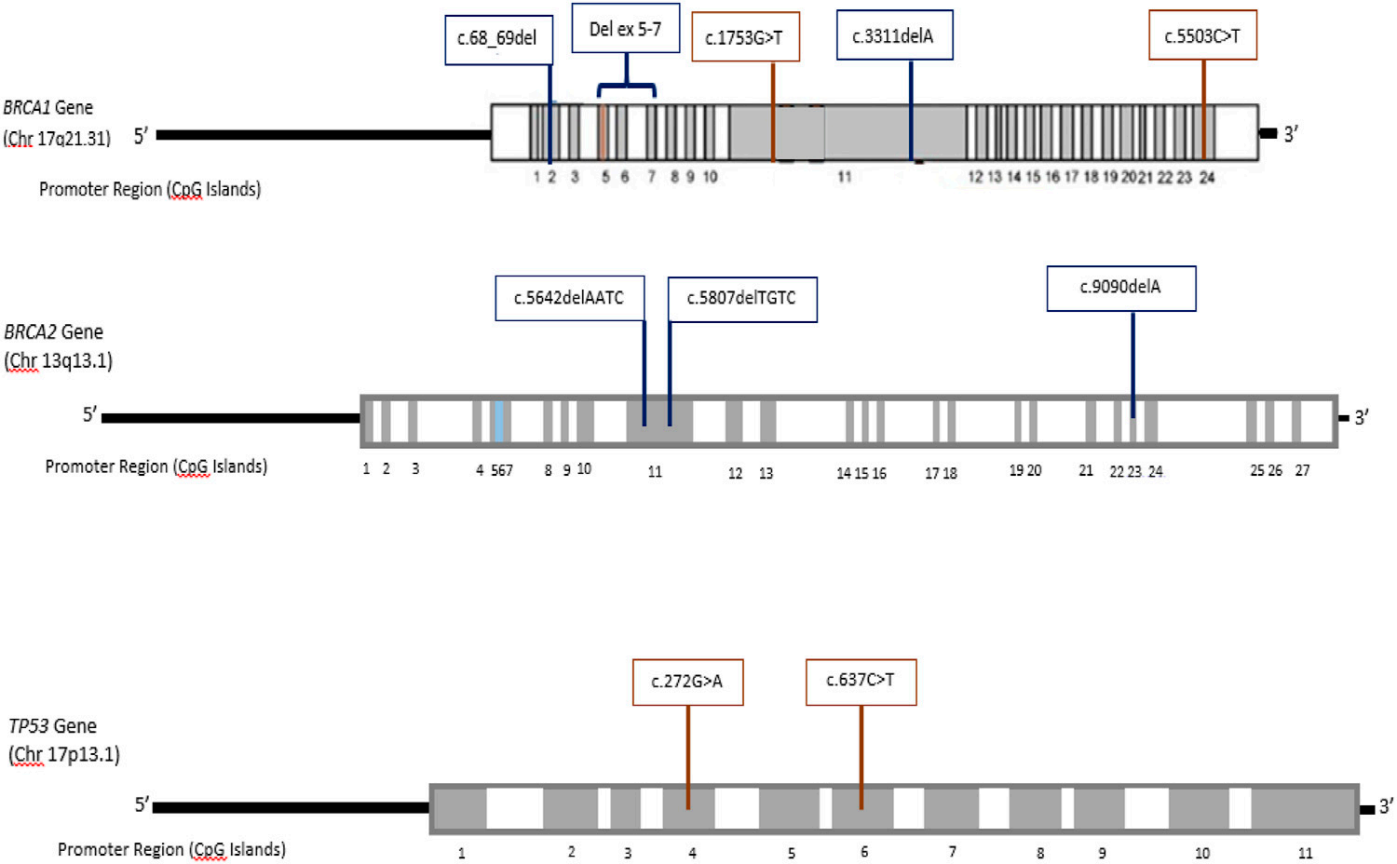
Exon-specific distribution of the identified germline mutations in *BRCA1*, *BRCA2*, and *TP53* genes.

In case of non-Caucasian females, there is paucity of data regarding molecular basis of breast cancers. To the best of our knowledge the present study is the first report of a twenty-seven breast cancer gene panel analysis in a South-Asian population. The molecular investigation includes high and moderate penetrance genes (Walsh et al., 2017). Here we report germ-line mutations in three high penetrance genes: *BRCA1*, *BRCA2,* and *TP53* in breast cancer patients from this population. The identified mutations consist of seven novel germ-line mutations, while three mutations have been reported previously. The location of inherited germ-line mutations the genes is shown in Figure 1.

The identified mutations were heterozygous. Bi-allelic *BRCA1* mutations are likely to be lethal at the embryonic stage, while such mutations in *BRCA2* lead to Fanconi anemia type D1, with increased risk of childhood cancer (FitzGerald et al., 1998; Moatter et al., 2011). Germline mutations in *TP53* are associated with Li-Fraumeni syndrome. In the present cohort, no syndromic cases were identified.

The founder mutations are expected in consanguineous populations like the present one. Although we did not find any recurrent mutation in the present cohort, comparison with previously published reports indicated that 185delAG may be a founder mutation (Liede et al., 2002; Rashid et al., 2006; Moatter et al., 2011; Ahmad et al., 2012; Aziz et al., 2016; Rashid et al., 2016; Rashid et al., 2017; Torres et al., 2017).

Germline mutations were not identified in the rest of reported high penetrant genes including *PTEN*, *CDH1,* and *STK11* (Boardman, 1998; Pharoah et al., 2001; Meijers-Heijboer et al., 2002; Lim et al., 2004; Tan et al., 2012).

Moderate penetrance genes include additional DNA repair genes. These are *CHEK2* (, *BRIP1* (*BACH1*), *ATM, PALB2* (Renwick et al., 2006; Seal et al., 2006; Rahman et al., 2007; Tanaka et al., 2012). These interact with *BRCA1* and/or *BRCA2*. The mutations in these genes result in two-fold increase in breast cancer risk. In the present study, no germ-line mutation was identified in these genes.

Similarly, no germline mutations were identified in other candidate genes *ATR, BARD1*, *FAM175A*, *FANCM*, *GEN1, MRE11A*, *NBN, RAD51B*, *RAD51C*, *RAD51D*, *RECQL, RINT1, SLX4, BAP1*, *XRCC2*, *CHEK1,* and *CTNNA1* (Varon et al., 1998; Stewart et al., 1999; Vahteristo et al., 2001; Bartkova et al., 2008; Turnbull et al., 2010; Loveday et al., 2011; Testa et al., 2011; Walsh et al., 2011; Wiesner et al., 2011; Loveday et al., 2012; Madanikia et al., 2012; Osher et al., 2012; Park et al., 2012; Ratajska et al., 2012; Solyom et al., 2012; Golmard et al., 2013; Majewski et al., 2013; Shah et al., 2013; Kiiski et al., 2014; Cybulski et al., 2015; Singh et al., 2018).

Interestingly, all the identified mutations are nonsense mutations and predicted to result in protein truncation. This corroborates the data for *BRCA1* and *BRCA2,* but not for *TP53* from India. Among South-Asian populations, the germ-line mutation rate (11.9%) in the present study is three folds less than reported for India (Mannan et al., 2016). In contrast to the reported observations that younger patients are likely to be the carriers of germ-line mutation, we report a lower frequency of such mutations as compared to the frequency of such mutations from India (36%).

Most studies on *BRCA1* and *BRCA2* mutations from Asia report a higher frequency for *BRCA2* mutations than *BRCA1*, the exceptions being Pakistan and India (Kim and Choi, 2013). The pattern is also observed in the present study.

The present study is also the first report of higher frequency of younger breast cancer patients belonging to Hindko and Sindhi ethnicities as compared to the other ethnicities in the region. As the investigated genes do not account for all such cases, it is possible that as yet unidentified gene(s) may be involved in these ethnic groups. It is pertinent to mention that the present day Pakistan consists of more than 12 distinct ethnic and linguistic groups (Mehdi et al., 1999; Qamar et al., 2002).

In conclusion, the present study while providing a framework for the investigation of genetic basis of breast cancers for cost-effective screening and management, raises many questions. The foremost is: as the germline mutations account for only 12% of the breast cancer cases, which other factors (genetic and/or environmental) are involved in the observed high incidence of breast cancers? It is expected that building on the present findings, a scientifically-focused approach may be developed for breast cancer research in a resource-limited setting.

## Data Availability

The original contributions presented in the study are included in the article/Supplementary Material, further inquiries can be directed to the corresponding author.
