# Supplementary material for: Germline Mutation Analysis in Sporadic Breast Cancer Cases With Clinical Correlations
Source: Front Genet. 2022 Mar 9;13:820610. doi: 10.3389/fgene.2022.820610 (PMC8959921; doi:10.3389/fgene.2022.820610)
Supplement: Supplementary file 1 [file Table1.pdf]

Supplementary Table S1. Clinicopathological Characteristics of the Breast Cancer Samples.

Sr. No.	*Tumour characteristic*	*Total number of samples*	*Value/ numbers*
**1.**	**Tumour Size**	**76**	**<2cm :** 10 (11.9%)
			**2-5cm :** 46 (54.8%)
			**>5cm :** 20 (23.8%)
**2.**	**Tumour Stage**	**58**	**T_1:** 03 (03.6%)
			**T_2:** 19 (22.6%)
			**T_3:** 32 (38.1%)
			**T_4:** 04 (4.8%)
**3.**	**Tumour Grade**	**80**	**G1:** 01 (1.2%)
			**G2:** 39 (48.7%)
			**G3:** 40 (50%)
**4.**	**Histopathology***	**84**	**IDC:** 68 (81%)
			**ILC:** 01 (1.2%)
			**DCIS + IDC:** 11 (13.1%)
			**Others:** 04 (4.80%)

*IDC: Invasive Ductal Carcinoma; DCIS: Ductal Carcinoma Insitu; In situ; ILC: Invasive Lobular Carcinoma
