# Supplementary material for: Germline Mutation Analysis in Sporadic Breast Cancer Cases With Clinical Correlations
Source: Front Genet. 2022 Mar 9;13:820610. doi: 10.3389/fgene.2022.820610 (PMC8959921; doi:10.3389/fgene.2022.820610)
Supplement: Supplementary file 2 [file Image1.pdf]

Supplementary Figure S1. Sequencing data for the identified germline mutations in *BRCA1*, *BRCA2,* and *TP53* genes.

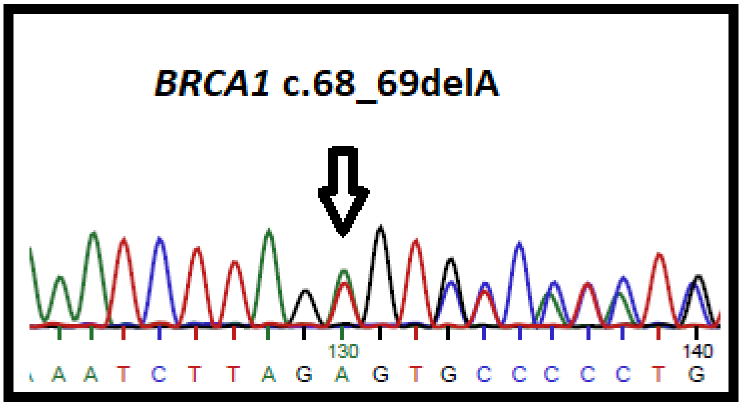

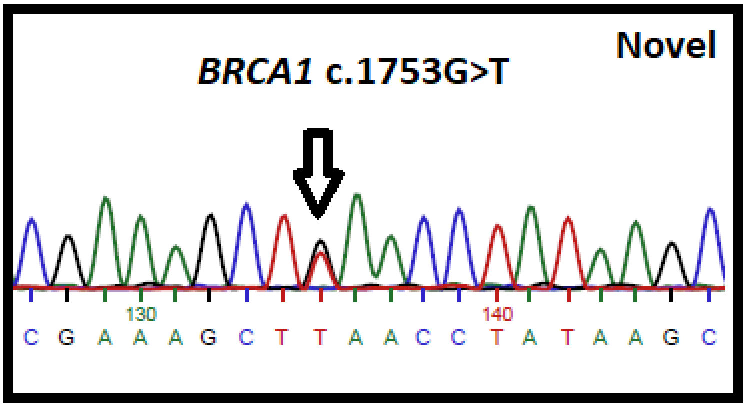

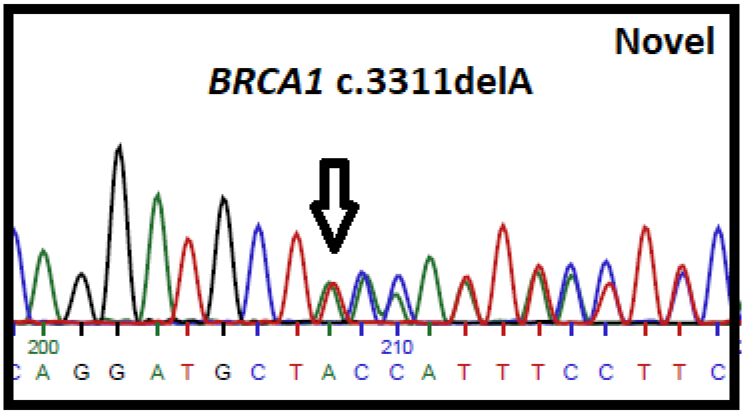

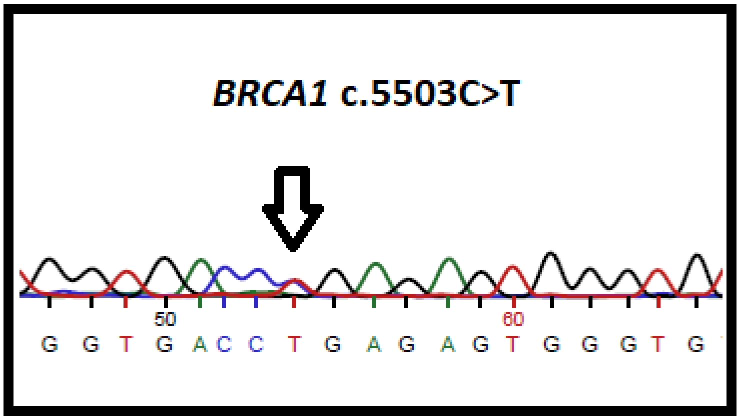
BRCA1 c.5503C>T
50
60
G G T G A C C T G A G A G T G G G T G

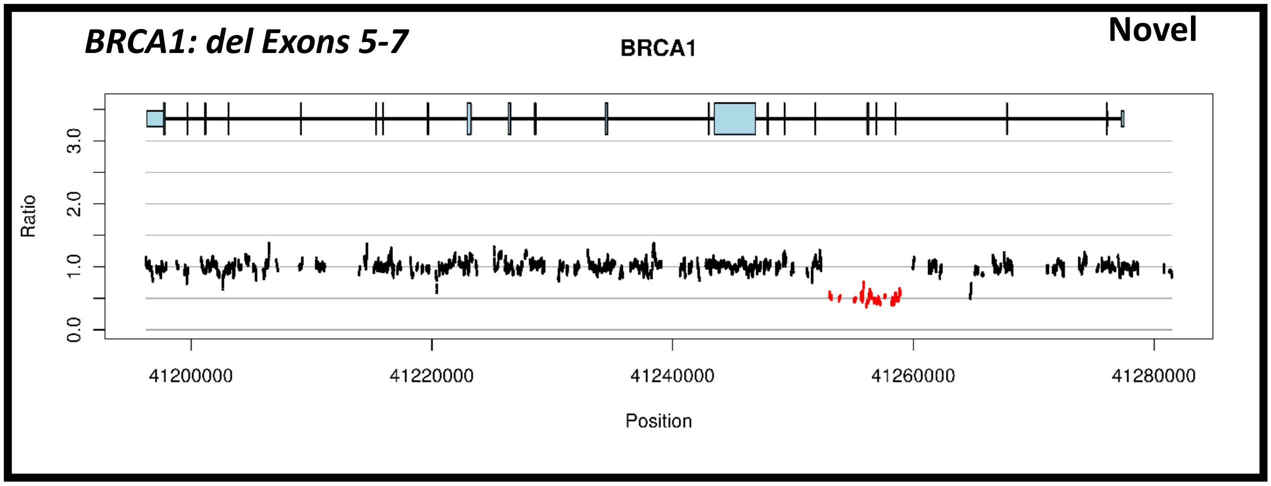
BRCA1: del Exons 5-7
BRCA1
Novel
Ratio
0.0
1.0
2.0
3.0
41200000
41220000
41240000
41260000
41280000
Position

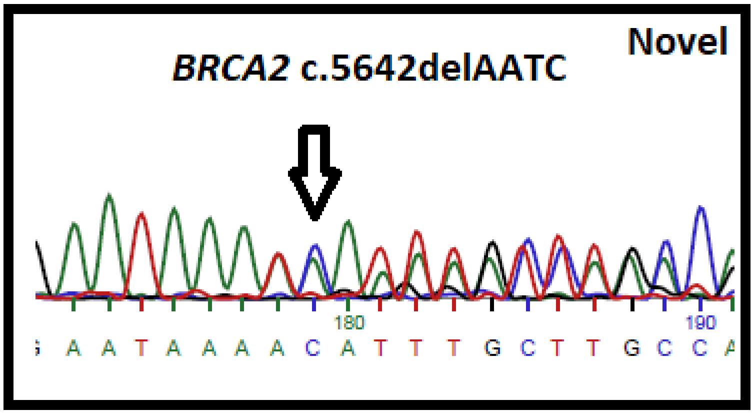
Novel
BRCA2 c.5642delAATC
180
190
A A T A A A A C A T T T G C T T G C C

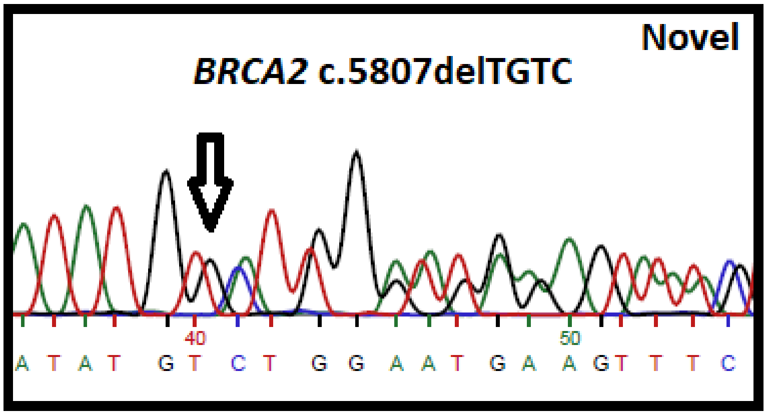
Novel
BRCA2 c.5807delTGTC
40
50
A T A T G T C T G G A A T G A A G T T T C

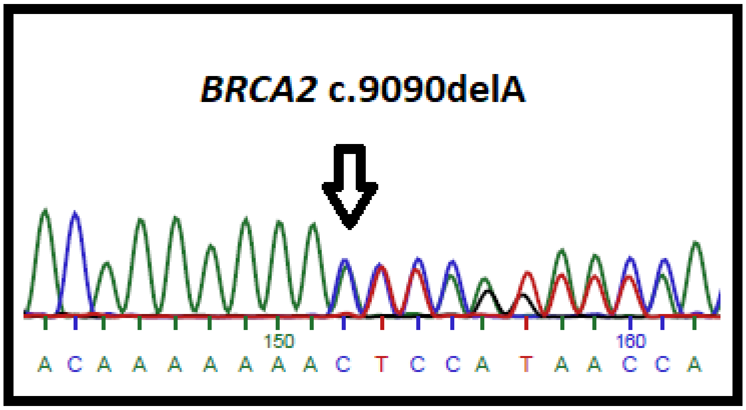
BRCA2 c.9090delA
150
160
A C A A A A A A A C T C C A T A A C C A

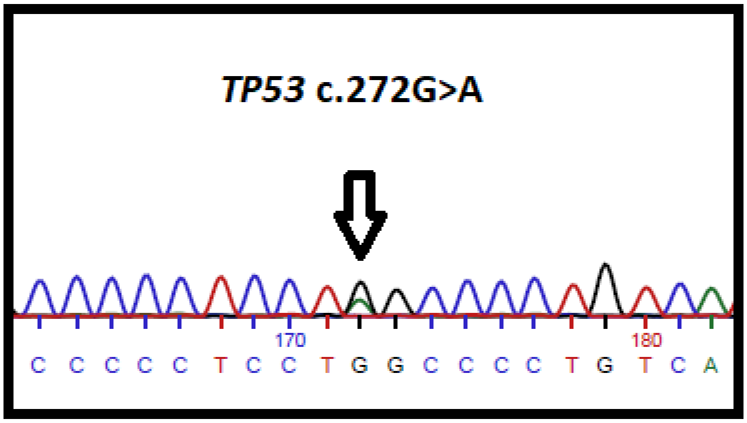
TP53 c.272G>A
170
180
C C C C C T C C T G G C C C C T G T C A

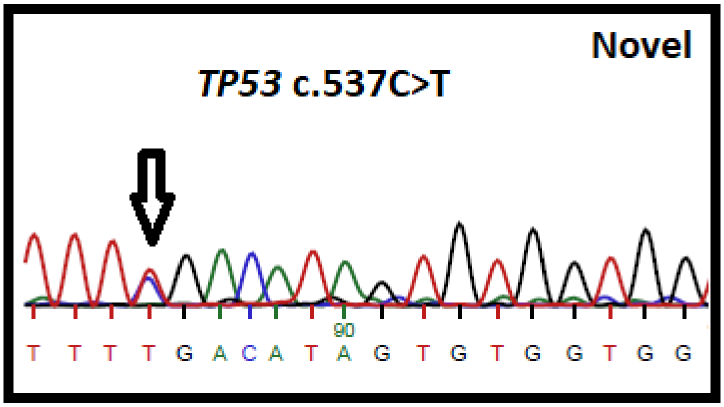

Supplementary Figure 1. Sequencing data for the identified germ-line mutations in *BRCA1*, *BRCA2*, and *TP53* genes.
